# Rural Versus Urban Distribution of Obstetrics and Gynecology Residency Training and Associated Rural Community Characteristics

**DOI:** 10.1111/jrh.70231

**Published:** 2026-10-19

**Authors:** Ryan Spencer, Mukesh Adhikari, Lori Rodefeld, Laney McDougal, AnnaMarie Connolly, Emily M. Hawes

**Affiliations:** ^1^ Department of Obstetrics and Gynecology University of Wisconsin School of Medicine and Public Health Madison Wisconsin USA; ^2^ Department of Health Policy and Management Gillings School of Global Public Health University of North Carolina at Chapel Hill Chapel Hill North Carolina USA; ^3^ The University of North Carolina Cecil G. Sheps Center for Health Services Research, University of North Carolina at Chapel Hill Chapel Hill North Carolina USA; ^4^ Wisconsin Collaborative for Rural Graduate Medical Education Sauk City Wisconsin USA; ^5^ Accreditation Council for Graduate Medical Education Chicago Illinois USA; ^6^ American College of Obstetrics & Gynecology Washington District of Columbia USA; ^7^ Department of Family Medicine University of North Carolina at Chapel Hill Chapel Hill North Carolina USA; ^8^ Eshelman School of Pharmacy, University of North Carolina at Chapel Hill Chapel Hill North Carolina USA

**Keywords:** health services, internship and residency, rural health

## Abstract

**Purpose:**

To assess the distribution of OB/GYN residency program training sites and compare community characteristics in rural counties with and without training sites.

**Methods:**

Using data from the Accreditation Council for Graduate Medical Education (ACGME), the locations of all accredited obstetrician/gynecologist (OB/GYN) residency training sites for the 2023–2024 academic year (AY 23–24) were geocoded. They were classified as rural or urban according to the Federal Office of Rural Health Policy and Core‐Based Statistical Area categories. The percentage of time spent training in rural areas was calculated for each program. Programs with at least 50% rural training were also identified. Bivariate analyses, including Pearson's chi‐square tests and Wilcoxon rank‐sum tests, were conducted using the Area Health Resource File (AHRF, 2022–23) to compare community characteristics between rural counties with and without OB/GYN residency training sites.

**Results:**

In AY 23–24, there were 294 OB/GYN residency programs utilizing 1052 training sites. There were 19 programs (6.5% of programs) offering a total of 25 training sites in rural areas (2.4% of all training sites). In programs that offered rural training sites, the rural experience accounted for an average of 4.8% of total training time in those programs. Regarding community characteristics, rural counties that did not have a residency training site were less populated than rural counties with a training site (15,742 vs. 50,886; *p* < 0.001). Compared with rural counties that had an OB/GYN training site, rural counties without a training site had a higher proportion of uninsured people less than 65 years old (8.0% vs. 11.5%; *p* < 0.001), a higher proportion of uninsured people less than 19 years old (3.8% vs. 6.4%; *p* < 0.001), lower median number of primary care physicians (40.6 vs. 55.1 per 100,000; *p* = 0.005), fewer median number of psychiatrists (0.0 vs. 2.5 per 100,000; *p* < 0.001), and were more likely for the entire county be a mental Health Professional Shortage Area (HPSA; 35.3% vs. 69.0%; *p*<0.001).

**Conclusion:**

OB/GYN residency training is highly urbanized. There are significantly less favorable health care access characteristics in rural counties without an OB/GYN residency training site compared to those with residency training sites. The findings identify rural communities without OB/GYN training sites that may represent opportunities for future rural training expansion. A comprehensive strategy to increase rural OB/GYN training experiences may increase the number of rural obstetricians.

## 1 Introduction

The Health Resources and Services Administration (HRSA) projects that the supply of obstetrician/gynecologists (OB/GYNs) will decrease by 3360 physicians by 2030 due to workforce retirement and work schedule allocation. This will coincide with an increase in demand such that there will be an estimated deficit of 5170 full‐time OB/GYNs by 2030 [1]. This deficit is likely to disproportionately affect rural communities, which already experience workforce shortages, recruitment challenges, and reduced access to obstetric services [2, 3]. As of 2022, roughly 59% of rural counties had no OB services even though the average number of births per patient is higher in rural areas [4, 5].

In 2022, 1052 counties in the United States did not have a health care facility providing OB care, and 2.2 million patients of child‐bearing age lost access to obstetrics services between 2020 and 2022. Multiple factors contribute to rural obstetric unit closures, including low birth volumes, workforce shortages, financial pressures, reimbursement challenges, and difficulties maintaining clinical staffing. Physician shortages, including shortages of OB/GYN physicians, family physicians providing obstetric care, and nursing staff, are frequently cited as important contributors to rural obstetric service loss [6, 7]. Conversely, increasing the OB/GYN workforce in rural areas by just 0.25 OB/GYNs per 1000 patients has been shown to be protective against rural OB unit closures [8, 9].

Although this study focuses on the OB/GYN workforce, maternity care in rural communities can be provided by a multidisciplinary workforce that includes family physicians and certified nurse‐midwives. These clinicians can also play a vital role in maintaining access to maternity care, particularly in communities where specialist availability is limited [10, 11].

The loss of OB services in rural areas impacts maternal and neonatal health outcomes. Kozhimannil et al. demonstrated that in the year following a rural OB unit closure, there was a significant increase in out‐of‐hospital births and preterm births in those counties [12]. Rural OB patients are at greater risk of postpartum hemorrhage and blood transfusions than urban patients and more than 50% of rural patients must travel greater than 30 min for OB care compared to 7% of urban patients [13].

Compounding these challenges, the United States is experiencing a rise in maternal mortality in rural locations. According to the Centers for Disease Control and Prevention (CDC), the maternal mortality rate increased to 32.9 deaths per 100,000 live births in 2021 from 20.1 per 100,000 live births in 2019 [14]. The risk of death in the peripartum period is twice as likely for rural patients than for urban patients and the gap between rural and urban maternal mortality is widening over time [15, 16].

Preventing further rural OB unit closures and the loss of services is crucial to avoid worsening maternal and neonatal outcomes in these areas. To achieve this, a comprehensive strategy focused on increasing the number of OB/GYNs practicing in rural locations is needed. Dedicated rural training in OB/GYN may be one component of a broader strategy to improve maternity care access in rural communities. Other factors influencing rural practice include educational debt, compensation opportunities, professional support, community characteristics, availability of obstetric services, and family considerations. Understanding the current distribution of OB/GYN residency training sites and identifying rural communities without training opportunities may help inform future workforce development efforts and identify opportunities for expansion of rural training experiences [17, 18].

The primary aim of this project was to identify and map the training sites for ACGME‐accredited OB/GYN residency programs and categorize them by rural or urban location. The secondary aim was to compare community characteristics between rural counties with and without OB/GYN residency training sites. Although rural residency mapping resources exist, this study provides a national assessment focused specifically on OB/GYN training sites and includes an analysis of rural community characteristics associated with training site distribution in this specialty.

## 2 Study Data and Methods

OB/GYN residency programs and their associated training site data were obtained from the ACGME's Accreditation Data System (ADS) for the AY 23–24. County characteristics were obtained from the AHRF (2022–2023).

Numerous definitions exist for classifying geographic locations as “rural” and “urban” [19]. Rural status was evaluated separately using two commonly used federal classifications: the Federal Office of Rural Health Policy (FORHP) rural definition and the Office of Management and Budget's Core‐Based Statistical Area (CBSA) classification. These definitions were not combined, and locations did not need to meet both definitions to be considered rural in the analyses. Results are reported separately for each classification because they are used for different federal rural health and graduate medical education (GME) policy purposes [20, 21]. The 2021 FORHP rural definition includes all non‐metro counties and census tracts within metropolitan counties that have Rural‐Urban Commuting Area (RUCA) codes of 4 or higher. According to the CBSA classification, counties are categorized as Metropolitan Statistical Area, Micropolitan Statistical Area, or neither. The latter two categories (micropolitan or neither) are designated as non‐metropolitan. These two rural definitions were selected because they are commonly used in US federal grant funding programs. The CBSA definition is used to determine whether a training site may qualify for additional Center for Medicare & Medicaid Services (CMS) payments for GME.

For residency data, we conducted analyses at two levels: training site level and program level. First, we geocoded training sites and determined their rural/urban status. Geocoding was performed using ArcGIS Pro 3.2.2 (Esri). We then merged the training site data with ACGME program codes and performed program‐level analysis. The percentage of rural training at the program level was calculated by dividing the total number of rotation months in rural sites by the standard number of months (48) for OB/GYN programs. Programs were classified as rural programs if at least 50% of their training occurred at rural sites.

To explore the county characteristics of training sites, we merged training site county data with AHRF data using county Federal Information Processing Standards (FIPS) codes. We compared county characteristics with or without training sites for rural counties. Categorical variables were compared using Pearson's chi‐square tests, and continuous variables were compared using Wilcoxon rank‐sum tests. A *p* value of < 0.05 was considered significant. All statistical analyses were performed using Stata, Version 18, for Mac (StataCorp, College Station, Texas).

## 3 Results

From the ACGME data, there were 294 OB/GYN residency training programs using 1052 training sites in AY 23–24. Table 1 summarizes program characteristics, regional distribution, and rural training exposure according to both FORHP and CBSA classifications. Of 294 OB/GYN residencies, 19 programs (6.5%) offered training in FORHP‐designated rural sites, and 14 programs (4.8%) offered training in CBSA non‐metropolitan sites. Only three programs offered at least 50% of training in FORHP‐designated rural locations and one program offered at least 50% of training in a CBSA non‐metropolitan location.

**TABLE 1:** Program characteristics and rural training exposure of obstetrics and gynecology residency programs using ACGME 2023–2024 data.

Variable	Category	All programs (*N* = 294)	Programs with FORHP rural rotations (*N* = 19)	Programs with CBSA non‐metro/micro rotations (*N* = 14)
Census region (*N*, %)	Midwest	71 (24.1%)	6 (31.6%)	5 (35.7%)
	Northeast	84 (28.6%)	3 (15.8%)	2 (14.3%)
	South	101 (34.4%)	9 (47.4%)	6 (42.9%)
	West	38 (12.9%)	1 (5.3%)	1 (7.1%)
Programs with rural rotations (N, %)	Yes	FORHP: 19 (6.5%)	—	—
	No	FORHP: 275 (93.5%)	—	—
Programs with non‐metro/micro rotations (N,%)	Yes	CBSA: 14 (4.8%)	—	—
	No	CBSA: 280 (95.2%)	—	—
Median % time in rural training		—	4.8 (4.2–14.6)	5.5 (4.2–14.6)
Programs with ≥ 50% rural training time	Yes	—	3 (15.8%)	1 (7.1%)
	No	—	16 (84.2%)	

*Note*: Data are presented as *n* (%) or median (IQR).

Abbreviations: ACGME, Accreditation Council for Graduate Medical Education; FORHP, Federal Office of Rural Health Policy; CBSA, Core‐Based Statistical Area.

Table 2 summarizes the characteristics and geographic distribution of the 1052 training sites used by OB/GYN residency programs. By either the FORHP or CBSA designations, only around 2% of all OB/GYN training sites are in rural areas (*n* = 25). This means that nearly 98% of all OB/GYN residency training takes place in training sites located in urban areas.

**TABLE 2:** Characteristics of training sites of obstetrics and gynecology residency programs using ACGME 2023–2024 data.

Variable	Category	Total *n* = 1052
Census region	Midwest	225 (21.4%)
	Northeast	226 (21.5%)
	South	404 (38.4%)
	West	197 (18.7%)
FORHP rural sites	Yes	25 (2.4%)
	No	1027 (97.6%)
Sites in CBSA non‐metro/micro counties (i.e., rural)	Yes	18 (1.7%)
	No	1034 (98.3%)

*Note*: Data are presented as *n* (%).

Figure 1 shows a map of the distribution of training sites throughout the United States. Areas where training is concentrated can be seen in the urban Northeast, populous areas throughout the South, the upper Midwest, and the California coast. The 25 sites that provide exposure to rural training are mostly in the Midwest and Appalachian areas. There are six states without OB/GYN residency training, and these include some of the most rural states in the country (Alaska, Idaho, Montana, North Dakota, South Dakota, and Wyoming).

**FIGURE 1:**
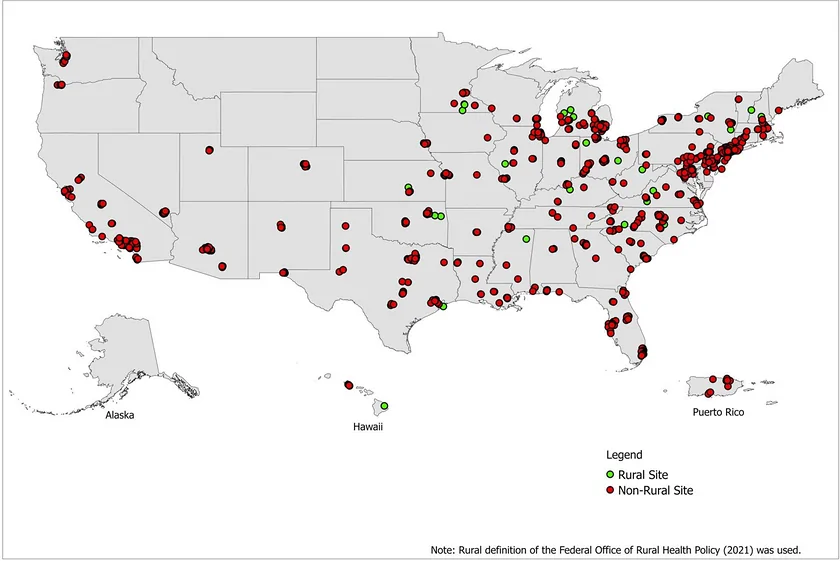
Geographic distribution of training sites of obstetrics and gynecology residency programs in the United States by rurality using ACGME 2023–2024 data.

Table 3 compares community characteristics between rural counties that do and do not have OB/GYN residency training sites. Those with training sites have a three times larger population base than those without training sites (median 50,886 vs. 15,742; *p* < 0.001). Rural counties with and without OB/GYN training are similar in the percentage of people below the federal poverty level and in racial demography, indicating similar population characteristics between the two groups that are common drivers of access and utilization of health care resources.

**TABLE 3:** Comparison of characteristics of non‐metro/micro (i.e., rural) counties with and without training sites using AHRF 2022–2023 data.

		Non‐metro/micro (i.e., rural) counties with training sites *N* = 17	Non‐metro/micro counties (i.e., rural) without training sites *N* = 1962	*p* value
Population estimate in 10,000 (2022)		5.1 (3.8–8.6)	1.6 (0.7–3.2)	< 0.001
Percent persons in poverty		14.5 (10.2–15.8)	14.5 (11.5–18.8)	0.22
% persons below poverty level		13.4 (9.5–15.8)	14.4 (11.0–19.0)	0.24
Percent Black/African American population		2.7 (1.7–5.1)	1.6 (0.8–5.9)	0.069
Percent Hispanic/Latino population		3.9 (2.7–7.9)	4.2 (2.4–9.3)	0.73
Percent White Hispanic population		3.4 (2.1–4.5)	3.2 (1.9–7.8)	0.71
% < 65 without health insurance		8.0 (6.6–8.9)	11.5 (8.5–15.8)	< 0.001
% < 19 without health insurance		3.8 (3.4–4.7)	6.4 (4.6–9.2)	< 0.001
Primary care physicians per 100,000 population		55.1 (46.0–76.9)	40.6 (23.4–60.4)	0.005
Psychiatrists per 100,000 population		2.5 (0.0–10.7)	0.0 (0.0–2.1)	< 0.001
HPSA primary care (2023)	Not	1 (5.9%)	168 (8.6%)	0.057
	Whole county	1 (5.9%)	604 (30.9%)	
	Part county	15 (88.2%)	1,183 (60.5%)	
HPSA mental health (2023)	Not	3 (17.6%)	72 (3.7%)	< 0.001
	Whole county	6 (35.3%)	1,349 (69.0%)	
	Part county	8 (47.1%)	534 (27.3%)	

*Note*: Data are presented as median (IQR) for continuous measures, and *n* (%) for categorical measures.

Abbreviations: AHRF, Area Health Resource File; HPSA, Health Professional Shortage Area.

The absence of OB/GYN training sites in rural counties is associated with a spectrum of less favorable health care access characteristics. The uninsured rate among the population under age 65 was higher in the counties without OB/GYN training (11.5% vs. 8.0 %; *p* < 0.001), as was the uninsured rate among the population under age 19 (6.4% vs. 3.8%; *p* < 0.001). Comparisons regarding access to health care providers were examined. The analysis shows that rural counties without OB/GYN training sites had significantly lower access to primary care physicians (PCPs) (median 40.6 PCPs/100,000 vs. 55.1/100,000; *p* = 0.005) and psychiatrists (median 0.0/100,000 vs. 2.5/100,000; *p* < 0.001) than rural counties with OB/GYN training sites.

Health Professional Shortage Areas (HPSAs) were also analyzed. The data show that rural counties without OB/GYN training sites are much more likely to be a mental health HPSA (96.3% of counties) versus rural counties with training sites (82.4% of counties). Additionally, rural counties without training sites were far more likely to have their entire county qualify as a mental health HPSA (69.0% of counties) than were rural counties with training sites (35.3%). Although not reaching statistical significance, it is notable that 30.9% of rural counties without training sites qualified as a primary care HPSA across the entire county.

## 4 Discussion

The findings demonstrate that OB/GYN residency training remains overwhelmingly concentrated in urban settings despite growing concerns regarding maternity care access in rural communities. This pattern mirrors broader trends across GME and highlights that physicians are predominantly trained in settings that differ substantially from the communities experiencing the greatest maternity care shortages. Nationally, the Government Accountability Office reported that only 2% of all residents receive any rural training experience, suggesting that the limited rural presence observed in OB/GYN is part of a broader challenge within graduate medical education [22].

Prior studies in family medicine demonstrate a strong association between rural training experiences and subsequent rural practice. Physicians who complete rural residency training are substantially more likely to practice in rural communities, and this association strengthens as the amount of rural training exposure increases [23]. Rural training exposure has been shown to be one of the strongest predictors of future rural practice, even when accounting for rural upbringing [23]. Although similar evidence is limited within OB/GYN because relatively few residents receive substantial rural training exposure, these findings suggest that expanding rural OB/GYN training opportunities may represent one component of a broader strategy to strengthen the rural obstetric workforce.

The development of formal rural OB/GYN training pathways has remained limited despite evidence from other specialties demonstrating the association between rural training experiences and subsequent rural practice. In the present study, only 1 programs offered any training in FORHP‐designated rural locations and only one program met the definition of a rural training program under the CBSA classification. In addition, only one ACGME‐designated OB/GYN Rural Track Program (RTP) existed nationally at the time of analysis. These findings illustrate the relatively slow growth of formal rural OB/GYN training pathways compared with the workforce needs of rural communities. This is a multifactorial issue that includes the ability to meet accreditation requirements, the recruitment and development of program leaders and faculty, and securing sustainable funding.

RTPs, generally partnerships between established urban training programs and smaller rural or community hospitals, require over 50% of residency training within a rural CBSA location to qualify for an increase in the CMS‐funded FTE cap for the urban partner [24]. Federal programs like the HRSA Rural Residency Planning and Development (RRPD) program can provide start‐up funding and technical assistance to establish new RTPs in OB/GYN [25]. Identifying rural hospitals with sufficient patient volumes to satisfy accreditation standards poses a challenge to further growth. Additional opportunities through initiatives such as the HRSA Teaching Health Center Graduate Medical Education program and the Centers for Medicare & Medicaid Services Rural Health Transformation Program are supporting rural training expansion in OB/GYN [26, 27, 28, 29].

Despite these obstacles, some OB/GYN programs have successfully leveraged alternative funding including Medicaid GME and state‐level funding for rural training experiences with less than 50% rural training time. The ACGME recognizes that there are several barriers to developing GME programs in rural and underserved areas and has taken steps to better understand them and identify ways to reduce them, such as through the major revisions to the Common Program Requirements process that is currently underway [30, 31]. To date, one OB/GYN program (in Moultrie, GA) has received the ACGME's RTP designation, which can offer a model to other programs interested in developing rural OB/GYN GME experiences [32].

In this study, OB/GYN rural training sites were more likely to be located in larger rural communities with greater physician workforce capacity and lower uninsured rates. These findings align with existing research suggesting that rural training opportunities may preferentially develop in communities with stronger health care infrastructure [33]. Conversely, many rural communities experiencing greater barriers to care currently have limited exposure to residency training programs. Because these analyses are cross‐sectional, it is not possible to determine whether training sites influence community characteristics or whether communities with stronger infrastructure are simply more capable of supporting residency training programs.

Exposure to populations with lower access to health services is a focus in OB/GYN training and practice—as it is for other specialties of medicine. These data show that there is a rich source of untapped potential for OB/GYN residency training in areas that lack access to insurance and other key health services across the spectrum of age. This includes patients in prime reproductive years—a focus of OB/GYN residency training. There are also significant opportunities to work with populations of patients who lack appropriate access to mental health and primary care services in rural locations that lack an OB/GYN residency training site.

In the analysis of the data regarding OB/GYN training site location and health services metrics, it is important to acknowledge potential limitations. These data are at risk for potential information bias. It is possible that not all programs have included all training sites in the ACGME ADS, which may result in the underreporting of rural opportunities or urban training sites for OB/GYN residency programs. Furthermore, the duration of training in each training site is self‐reported and may not be accurate or apply to all trainees in the program. As only a single academic year was analyzed, we could not capture any changes in the rural–urban distribution of training sites and program characteristics over time. Additionally, we analyzed health services characteristics by all training sites rather than just the primary training site of each program. This decision was made to perform a more comprehensive analysis given the paucity of programs that offer training in rural locations. Training site data provided the greatest understanding of the differences in characteristics among all locations that support some degree of OB/GYN residency training. OB/GYN training is not the only type of training program that may be associated with these and other, lower relative levels of health services. Causality should not be inferred or assumed.

Even so, medical training environments can be a nidus for additional health care services and resources such as primary care and access to mental health providers. The root cause for individual HPSAs, whether for primary care or mental health, is complex and multifactorial. The goal of these analyses is not to say that the absence of OB/GYN training is a factor that leads to these poorer health service characteristics. Rather, it highlights the association that exists. These data may serve to motivate the OB/GYN medical education community to holistically discuss and analyze the degree to which investment in new rural training sites will expose future OB/GYN physicians to the challenges that patients encounter in these locations.

Another advantage in developing a comprehensive strategy to proactively engage in increasing OB/GYN training in rural sites is the opportunity for residents to learn and understand how to provide care in settings with less resources. Rural areas are those hardest hit by physician attrition and are challenged in physician recruitment. It is known that increasing exposure to training in rural locations increases the likelihood of practicing in rural areas after training.

Expanding rural OB/GYN training opportunities is unlikely to fully address the complex causes of rural maternity care shortages. Understanding where training currently occurs, identifying gaps in rural training exposure, and developing partnerships with rural communities capable of supporting educational experiences may help inform broader workforce development efforts. Rural training should be considered one component of a comprehensive strategy that also addresses financial sustainability of obstetric services, recruitment and retention of clinicians, infrastructure needs, and broader rural health system challenges.

## Funding

The Rural Residency Planning and Development Program–Technical Assistance Center (RRPD‐TAC) is supported by the Health Resources and Services Administration (HRSA) of the U.S. Department of Health and Human Services (HHS) under cooperative agreement #UK6RH32513. The Teaching Health Center Graduate Medical Education Technical Assistance Center (THCGME‐TAC) is supported by the HRSA of the U.S. Department of HHS under contract #75R60224C00016.

## Disclosure

The contents are those of the authors and do not necessarily represent the official views of, nor an endorsement, by HRSA, HHS, or the US Government.

## Ethics Statement

The study received an exemption determination from the Institutional Review Board (University of North Carolina Institutional Review Board 22‐0425).

## Conflicts of Interest

The authors declare no conflicts of interest.

## Data Availability

The data that support the findings of this study are available on request from the corresponding author. The data are not publicly available due to privacy or ethical restrictions.
